# *Sargassum fusiforme* polysaccharides modulate gut microbiota and metabolites to regulate hyperlipidemia in mice fed a high-fat diet

**DOI:** 10.1128/aem.01445-25

**Published:** 2025-11-11

**Authors:** Ning Su, Shiwei Han, Zhengyang Li, Xiaoyu Ling, Lingqing Kong, Dafeng Song

**Affiliations:** 1 Key Laboratory for Food Microbial Technology of Zhejiang Province, School of Food Science and Biotechnology, Zhejiang Gongshang University 12625, Hangzhou, Zhejiang, China; Universita degli Studi di Napoli Federico II, Portici, Italy

**Keywords:** *Sargassum fusiforme*, lipid lowering, gut microbiota, lipid metabolism

## Abstract

Hyperlipidemia complications caused by obesity are a hot issue threatening human health worldwide, and there is an urgent need to explore low-toxicity health foods for intervention. *Sargassum fusiforme* polysaccharides (SFPS) display cholesterol-lowering properties, but the underlying mechanism has not been elucidated. This study investigates the mechanisms by exploring changes in gut microbiota composition, gene expression, and metabolites in mice fed a high-fat diet after intervention with *S. fusiforme* fucoidan (SFF) and SFPS through 16S rRNA sequencing, transcriptomics, and non-targeted metabolomics. The experimental findings indicated that SFPS markedly enhanced the abundance of Akkermansia while concurrently reducing the levels of Actinobacteria, Erysipelotrichaceae, Bifidobacteriaceae, and Peptostreptococcaceae. This was achieved by upregulating the expression of Mt1, Prlr, and slc25a21 and downregulating SREBP-1, thereby inhibiting the cholesterol metabolism pathway. These changes resulted in increased hepatic production and fecal excretion of bile acids and reduced hepatic cholesterol. Our results shed light on the mechanisms behind the cholesterol- and lipid-lowering effects of SFPS, suggesting its potential as a therapeutic agent for hypercholesterolemia.

**IMPORTANCE:**

Obesity and its associated metabolic disorders pose a global health challenge, necessitating safe and scalable interventions. This study demonstrated that polysaccharides from *Sargassum fusiforme* (SFPS), extracted via a novel non-alcoholic precipitation method, effectively ameliorate high-fat diet (HFD)-induced obesity by remodeling gut microbiota and restoring metabolic homeostasis. Integrating multi-omics approaches, we reveal that SFPS enriches beneficial taxa like Akkermansia while suppressing obesity-linked bacteria like Actinobacteria, Erysipelotrichaceae, and Bifidobacteriaceae; modulates cholesterol metabolism through gene regulation (e.g., downregulating Srebf1 and upregulating Mt1); and enhances bile acid excretion. Notably, SFPS exhibits efficacy comparable with the well-studied fucoidan (SFF); however, its cost-effective extraction method offers superior scalability for functional food development. These findings underscore the potential of SFPS as a prebiotic agent targeting the gut-liver axis, providing mechanistic insights into natural product-based strategies for metabolic disease management. This work advances our understanding of how polysaccharides interact with the host microbiome and metabolism, advancing dietary interventions for obesity management one step further.

## INTRODUCTION

Obesity has become a global public health problem (1). According to the data from the World Health Organization, at least 2.8 million people die each year from complex diseases caused by obesity and overweight, among which hyperlipidemia is included. The change in dietary habits, which is the increase in high-fat food intake and a sedentary lifestyle, is the main reason for the prevalence of hyperlipidemia (2). At present, the most effective methods to prevent and treat hyperlipidemia are a low-fat diet and exercise. However, over the long term, the effectiveness of lifestyle interventions aimed at reducing energy intake and increasing energy expenditure is limited because the complex and continuous hormone secretion, neurometabolism, and other regulatory mechanisms of the body will prevent weight loss and promote weight regain (3). Therefore, the researchers studying weight loss have focused on identifying healthy foods to prevent and treat hyperlipidemia from natural products in recent years.

*Sargassum fusiforme* belongs to the Sargassaceae plant of the phylum brown algae. It is a common edible seaweed in Eastern Asian countries and regions. It is characterized by high polysaccharide content, low-fat composition, low-calorie properties, and abundant mineral content. Therefore, it is called “longevity vegetable” and “sea ginseng on the table” by many island and coastal residents (4). The biological activities of *S. fusiforme*, such as lowering blood fat (5), lowering blood sugar, anti-tumor, anti-oxidation, anti-inflammatory, and regulating immunity, are closely related to its polysaccharide (6). *S. fusiforme* polysaccharides (SFPS) are the main components of *S. fusiforme*. The polysaccharides of *S. fusiforme* mainly include fucoidan, alginic acid, and laminaran. Fucoidan from *S. fusiforme* is a sulfated heteropolysaccharide containing fucose. It has various physiological functions and is an important bioactive substance in *S. fusiforme*. Fucose-containing substances can regulate the intestinal microbiota (7–9). According to studies, the fucoidan from *S. fusiforme* can change the structure of intestinal microbiota in mice, which is a potential mechanism to alleviate inflammation and diabetes (10).

The gut is a complex ecosystem with a large microbial community, called the gut microbiota, which is closely related to the host. The hosts co-evolved to form a mutually beneficial symbiotic relationship (11). The large number of human gut microbes plays an important role in regulating obesity caused by a high-fat diet, and dysbacteriosis will lead to obesity (12). Studies demonstrate that obesity correlates with shifts in the Firmicutes/Bacteroidetes (F/B) ratio, with the F/B ratio decreasing during weight loss remission (13). Critically, high-fat diets (HFD) exacerbate this dysbiosis, impairing intestinal barrier function and elevating systemic lipopolysaccharides. This triggers chronic inflammation and insulin resistance, which are central drivers of lipid metabolism dysregulation and hyperlipidemia (14). Therefore, regulating the structure of intestinal flora and maintaining the dynamic balance of intestinal microecology are the key entry points for the treatment of lipid metabolism dysregulation and hyperlipidemia.

In this study, Sargassum served as the raw material, and Sargassum polysaccharides were extracted using the ceramic membrane concentration method. Ethanol precipitation is conventionally recognized as a primary technique for polysaccharide extraction, applicable to nearly all water-soluble polysaccharides. Despite its relative simplicity, this method requires multiple rounds of reprecipitation to improve polysaccharide purity, which may lead to substantial losses and decreased recovery rates. Conversely, the ceramic membrane concentration method represents an efficient and energy-saving technology for separation and purification in polysaccharide extraction, widely employed in the extraction of plant and algal polysaccharides. In this research, after acquiring the polysaccharide solution, ceramic membranes were utilized as an alternative to the traditional ethanol precipitation method. This allowed small molecules to pass through the membrane while retaining larger molecules like polysaccharides, thereby achieving concentration. The process avoided the high-temperature conditions typically associated with traditional concentration methods, thereby maximizing the preservation of the biological activity of polysaccharides and eliminating the need for organic solvents (e.g., ethanol), thus reducing pollution and aligning with the principles of green production.

In this study, the widely studied SFF was used as a control. By studying the changes in gut bacteria and metabolites after the intervention of SFPS, as well as the differences in gene expression after SFPS intervention, the potential mechanism by which SFPS improves hyperlipidemia is explored.

## MATERIALS AND METHODS

### Materials

*S. fusiforme* (SF) was purchased from the Zhejiang Wenzhou Dongtou supermarket. Fucoidan extracted from *S. fusiforme* was purchased from the Shandong Jiejing group. Other reagents and solvents were of analytical grade.

### Extraction of crude polysaccharides from *S. fusiforme*

Fresh SF was soaked in a citric acid solution at 75°C for 3 h and subjected to ultrasound treatment for arsenic removal. The ultrasound-treated SF was washed, crushed, and ground into a homogeneous slurry. Distilled water was added to the slurry at a solid-liquid ratio of 1:8, and the mixture was extracted three times in a 50°C water bath. A zirconia membrane with a molecular weight cutoff of 100 kDa was selected to filter and retain the polysaccharides of *S. fusiforme*. The extracts were collected, and the filtrate was concentrated using a vacuum rotary evaporator for 10 min. The concentrated sample should be freeze-dried into powder and subsequently collected for future use. The molecular weight distribution was characterized by high-performance size-exclusion chromatography (HPSEC) coupled with multi-angle laser light scattering (MALLS) and differential refractive index detection (RI). Specifically, a DAWN HELEOS III detector (Wyatt Technology) was employed in conjunction with a TSC-GEL G4000PWxl series column (Tosoh Bioscience), using 0.1M NaNO₃ as the mobile phase at a flow rate of 0.5 mL/min. Sample concentration was maintained at 1 mg/mL, and the molecular weight data were calculated using Astra 8 software with a dn/dc value of 0.146 mL/g. Elemental composition analysis for carbon (C), hydrogen (H), nitrogen (N), and sulfur (S) was performed directly via combustion using a PerkinElmer EA 2400 II elemental analyzer. Oxygen (O) content was derived by mass difference subtraction according to the formula %O = 100% - (%C + %H + %N + %S + %Ash), where %Ash represents an independently determined ash content. The infrared spectra were recorded in the wavenumber range of 4,000–400 cm^−1^ using a Nicolet iS5 Fourier transform infrared spectrometer (Thermo Fisher Scientific, Germany).

### Animal and experimental design

Forty male C57BL/6NCr mice (Specific-pathogen-free grade, 20 ± 2 g, 5-week-old) were purchased from Zhejiang Yingyang Biotechnology Co., Ltd. All mice were randomly divided into four groups, with each group consisting of 10 animals and fed a normal diet for 1 week to stabilize all the metabolic conditions. Each group was housed in one standard cage under conditions of 22 ± 2°C, a humidity of 60 ± 5%, and a 12 h light/dark cycle. The specific grouping is as follows: ND group, fed with normal diet (low-fat D12450J), gavaged with 200 µL deionized water; HFD group, fed with high-fat diet (high-fat D12492), gavaged with 200 µL deionized water; HFD + SFF group, fed with high-fat diet, gavaged with 200 µL fucoidan; and HFD + SFPS group, high-fat diet, intragastric administration of 200 µL polysaccharide.

During the experiment, the body weight was recorded every 3 days. After 8 weeks of SFF and SFPS intervention, all mice were euthanized by cervical dislocation after blood collection from the eyeballs. Blood samples were centrifuged to obtain the plasma, which was immediately stored in a −80°C freezer. The mice were dissected, and the liver, epididymis, epididymal fat, colon, and feces were collected. Fecal samples were immediately frozen at −80°C, whereas the liver and epididymis were fixed in 10% formaldehyde for cross-sectional analysis. The remaining liver samples were immediately placed in liquid nitrogen and transferred to a −80°C freezer for subsequent analysis of gene expression levels. All other collected samples were stored in a −80°C freezer.

### H&E staining

The freshly isolated liver was placed into 4% paraformaldehyde to fix for 48 h and then dehydrated with different concentrations of ethanol, followed by embedding in paraffin. The specimens were then serially sliced into 4-µm thick slices and stained with hematoxylin and eosin (H&E). Histological images were obtained using an optical microscope (Nikon, Japan). Five different visual fields from each section were selected, and the number of adipocytes was measured using ImageJ software.

### Short-chain fatty acids (SCFAs) quantification

Gas chromatography–mass spectrometry (GC-MS) is the most widely used and reliable method for SCFA analysis in mouse feces (15). The specific steps are as follows: take 50 mg of the fecal sample stored at −80°C and add 0.2 mL of 0.5% (vol/vol) phosphoric acid solution. After grinding, the mixture was vortexed for 10 min for thorough mixing. 500 µL of MTBE solvent containing internal standards was added, followed by vortexing for 3 min and sonication under an ice bath for 5 min. The mixture was centrifuged at 12,000 rpm and 4°C for 10 min. After centrifugation, the upper clear liquid (200 µL) was transferred to an injection vial with a glass liner for GC-MS/MS analysis (DUIS-GCMS-QP2020NX).

### Determination of bile acid

The experiment used LC-MS/MS to determine bile acids in mouse biological samples (16). In total, 20 mg of solid sample was added to 200 µL of methanol containing internal standards for homogenization. After homogenization, shake the sample at 2,500 rpm for 10 min, then place it at −20°C for 10 min. Centrifuge at 12,000 rpm for 10 min and collect the supernatant for concentration. The concentrated sample is reconstituted with 50% methanol-water for LC-MS/MS analysis (DUIS-LC-MS-2020).

### Blood lipid parameter

The content of total cholesterol (TC) and triglycerides (TG) in the serum was measured using assay kits from Jiancheng Bioengineering Institute (Nanjing, China). The results were read using an enzyme standard instrument (Molecular Devices, SpectraMax i3, Shanghai, China).

### PCR amplification and 16S rRNA sequencing

Small fragment libraries were constructed based on the characteristics of the PCR-amplified 16S region (10). The library was then subjected to paired-end sequencing on the Illumina NovaSeq platform. After filtering and assembling the reads, representative sequences were generated using clustering or denoising methods. Species annotation and abundance analysis were performed on the representative sequences. Alpha and Beta diversity analyses were conducted to reveal differences in species composition and community structure among the samples.

### Analysis of gene expression levels

The quality of the extracted RNA from the samples was assessed using agarose gel electrophoresis, Qubit 2.0 fluorometer, and Agilent 2100 Bioanalyzer. After confirming the quality of the RNA, libraries were constructed. The effective concentration of the libraries was quantified using Q-PCR to ensure accurate measurement (library effective concentration >2 nM). Once the library quality met the requirements, the libraries were pooled based on the desired sequencing depth, and sequencing was performed on the Illumina platform.

### Untargeted metabolomics study

Fifty milligrams of feces were mixed with 70% methanol water. After thorough mixing, the mixture was centrifuged at 4°C and 12,000 rpm for 10 min, and the supernatant was collected. Ethyl acetate/methanol (vol/vol, 1:3) was added to the pellet, and then the centrifugation steps described above. The supernatants were combined and concentrated. The dried residue was reconstituted with 70% methanol-water, subjected to ultrasound, and centrifuged. 60 µL of the supernatant was transferred to a glass insert tube for LC-MS/MS analysis.

### Comprehensive targeted metabolomics analysis

Serum samples were vortexed, centrifuged, and 50 µL of the sample was mixed with 1 mL of lipid extraction solution (isobutyl methyl ketone: methanol = 3:1, vol/vol, containing internal standard mixture). The mixture was vortexed, sonicated, and then vortexed for 1 min after adding 200 µL of water. The mixture was centrifuged at 12,000 rpm and 4°C for 10 min; 500 µL of the supernatant was collected and concentrated. The concentrated sample was reconstituted with mobile phase B for LC-MS/MS analysis.

### Statistical analysis

The Quantitative Insights into Microbial Ecology (QIIME, version 1.9.1) pipeline was employed for the analysis of sequence data, wherein valid sequences were discerned through the elimination of low-quality reads. An operational taxonomic unit (OTU) was established, which included processes such as dereplication, clustering, and chimera detection utilizing the V search tool. To study the species composition of each sample, OTU clustering was performed on the valid data of each sample based on the principle of 97% sequence similarity. Additionally, OTU-level alpha diversity indices were calculated using QIIME and R packages (v4.1.2). Beta diversity was assessed utilizing UniFrac distance metrics and visualized via Non-Metric Multi-Dimensional Scaling (NMDS). Intergroup differences in species diversity were assessed using either the *t*-test (for comparisons between two groups) or the Kruskal-Wallis test (for comparisons involving three or more groups). In non-targeted metabolomics, the screening of differential metabolites is primarily based on the VIP (variable importance in projection) values derived from the OPLS-DA model (with biological duplicates), where metabolites with VIP >1 are selected. Additionally, the results of univariate statistical analysis with a *P*-value threshold of less than 0.05 are incorporated to further refine the selection of differential metabolites. To ensure sample preparation uniformity, quality control (QC) samples were interspersed throughout the analytical sequence. This approach allowed assessment of reproducibility across both sample preparation and instrumental analysis processes. QC performance was monitored using principal component analysis (PCA) to ensure consistent analytical stability throughout the run. For GO enrichment analysis, clusterProfiler (Version 4.6.0) was employed, with bubble charts generated for visualization purposes.

## RESULTS

### Analysis of the main components of SFPS

The SFF contained carbohydrate (92.61% ± 2.41 %), protein (1.25% ± 0.02 %), uronic acid (27.81% ± 0.23 %), and sulfate (5.08% ± 0.33%). Based on molecular weight analysis data, SFF was a heterogeneous polysaccharide consisting of two fractions with 201.4 kDa (88.32%) and 62.5 kDa (11.68%). Organic element analysis revealed that the mass ratios of C, H, O, N, and S were 33.34%, 6.67%, 46.75%, 0.22%, and 2.45%. The SFPS contained carbohydrate (63.28% ± 3.26%), protein (12.41% ± 1.76%), uronic acid (18.52% ± 0.19%), and sulfate (4.49% ± 0.43%). Molecular weight distribution analysis by HPSEC demonstrated SFPs to be a highly polydisperse mixture, containing high-molecular-weight aggregates (>300 kDa) and low-molecular-weight fractions (<50 kDa) attributable to protein-polysaccharide complexes and salts. Organic element analysis revealed that the mass ratios of C, H, O, N, and S were 26.14%, 5.08%, 38.87%, 3.92%, and 1.85%.

Based on Fourier transform infrared spectroscopy (FTIR) analysis (Fig. S1), the polysaccharides isolated from *S. fusiforme* in this study exhibit structural features characteristic of fucoidan. A broad absorption band near 3,400 cm^−1^ is attributed to the O-H stretching vibration, indicating the presence of abundant hydroxyl groups. The weak peak at 2,920 cm^−1^ corresponds to C-H stretching vibrations of methylene groups in the sugar ring. A strong absorption peak at 1,250 cm^−1^ confirms the presence of sulfate ester groups (asymmetric S = O stretching), whereas a characteristic absorption at 820 cm^−1^ suggests that sulfate substitution predominantly occurs at the C4 position of fucose residues, consistent with axial orientation. The minor absorption observed at 1,650 cm^−1^ may arise from either the C = O stretching vibration of trace uronic acid or residual moisture. These findings collectively indicate that the polysaccharide from *S. fusiforme* is a highly sulfated fucoidan featuring an α-linked fucose backbone with predominant sulfation at the C4 position and potential minor incorporation of uronic acid moieties.

### Effects of SFPS on body weight in HFD-fed mice

**Fig 1:**
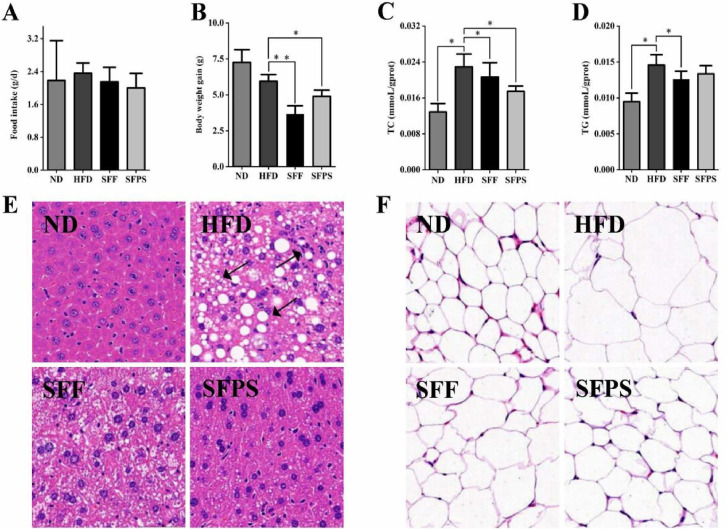
(**A**) Histogram of the food conversion of mice in each group. (**B**) Histogram of the body weight of mice in each group. (**C**) Histogram of the TC of mice in each group. (**D**) Histogram of the TG of mice in each group. (**E**) Liver sections of mice in each group. (**F**) Epididymal assections of mice in each group, *P < 0.05, **P < 0.01.

C57BL/6 N mice subjected to an HFD were administered 400 mg/kg of SFPS for 8 consecutive weeks, and the effects of SFPS and SFF on the body weight of mice were observed. At the conclusion of the animal experiments, body weight in the HFD group exhibited a significant increase; however, supplementation with SFPS and SFF markedly mitigated body weight gain (BWG) associated with HFD (Fig. 1B). Importantly, there were no statistically significant differences in mean food intake among the groups (Fig. 1A).

### Effects of SFPS on serum lipid profiles and liver histopathology in HFD-induced hyperlipidemic mice

At the end of the animal experiments, HFD feeding led to a significant increase in serum TC and TG (Fig. 1C and D). Compared with the ND group, the SFPS and SFF groups reduced TG and TC levels. Besides, compared with the ND group, SFPS supplementation significantly decreased these indices. Although the SFF has a more obvious decrease in many blood lipid indicators, the effect of SFPS almost reaches that effect of SFF. The above findings demonstrated that SFPS could attenuate the indices of HFD-induced hyperlipidemia.

H&E staining of the ND group revealed a well-organized and tightly arranged hepatic lobule structure. In contrast, the HFD group displayed significant lipid droplet accumulation, inflammatory cell infiltration accompanied by focal necrosis, and ballooning degeneration (Fig. 1E). Notably, these pathological alterations were mitigated following SFPS treatment. Additionally, histological H&E staining was conducted on epididymal adipose tissues to assess the inhibitory effects of SFF and SFPS on lipid accumulation in these tissues. Histological images presented in Fig. 1F indicate that the area occupied by white adipocytes was markedly increased in the HFD group compared with the ND group. Conversely, there was a substantial reduction in white adipocyte area within both SFF and SFPS groups. These findings suggest that 4 weeks of HFD intervention induced hyperlipidemia; however, supplementation with SFPS effectively attenuated lipid accumulation in both epididymal adipose tissues and hepatic tissues.

### SFPS modulated gut microbial community composition at different taxonomic levels

**Fig 2:**
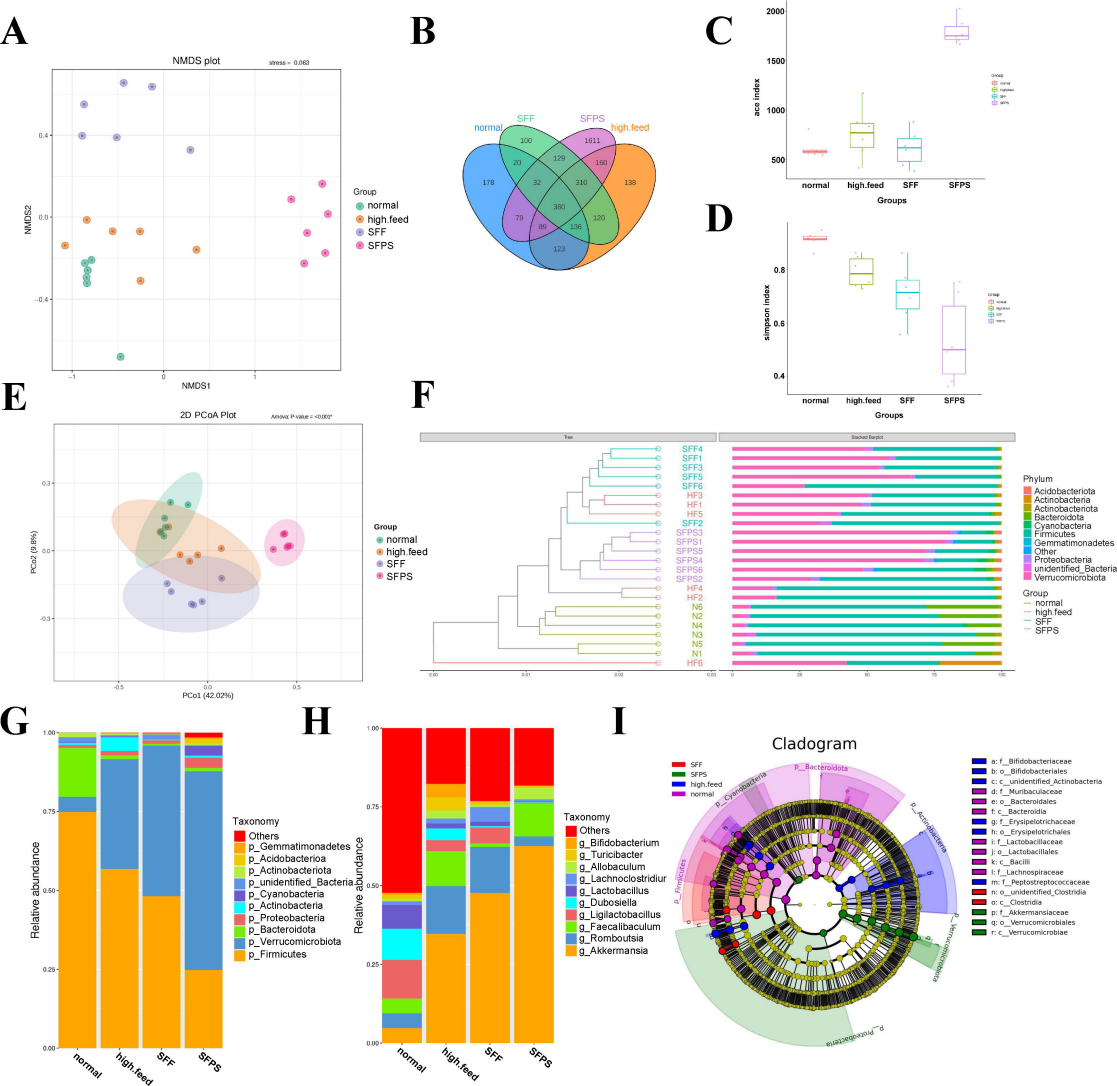
Intestinal microbiota profile and differential microbial community screening. (**A**) Non-metric multidimensional scaling (NMDS) analysis based on operational taxonomic unit (OTU). (**B**) Venn diagram analysis of differential pathways. (**C**). Box plot showing differences in the ACE index based on OTU. (**D**) Box plot showing differences in the Simpson index based on OTU. (**E**) PCoA based on OTU abundance. (**F**) Unweighted pair group method with arithmetic mean (UPGMA) clustering tree based on OTU weighted Unifrac distance. The left side shows the UPGMA clustering tree structure, and the right side displays the relative abundance distribution map of each sample at the phylum level. (**G**) A stacked bar chart illustrating the relative abundance of species categorized at the phylum level across various groups, as determined by operational taxonomic units (OTUs). (**H**) Stacked bar chart of species relative abundance at the genus level for different groups based on OTU. (**I**) Evolution branch graph based on OTU.

Gut microbes are crucial for dietary metabolism. We investigated whether SFPS influences fat regulation by analyzing 16S rRNA in fecal samples from various dietary groups (N, HF, SFF, and SFPS). Using the NMDS method based on OTU data, we observed a clustering trend within groups, but no significant clustering patterns in the N, HF, SFPS, and SFF groups. Because a high-fat diet also had an impact on the SFF and SFPS groups, interfering with the composition of their metabolites (Fig. 2A).

The total number of OTUs in the HFD group is lower compared with the normal diet group, whereas SFPS supplementation reversed such a decline (Fig. 2B). SFPS supplementation significantly increased the Ace indexes (**P < 0.01) and decreased the Simpson index, compared with the HFD group, indicating that SFPS improves the diversity and richness of the microbial community (Fig. 2C and D). PCoA based on OTU abundance further revealed that gut microbial composition exhibited an apparent response to HFD and SFPS interventions (Fig. 2E). The gut microbial composition in the HFD group was not completely separated from that of the ND group. Although the aggregation of the SFF group was close to that of the HFD group, the SFPS group was distinctly separated from the HFD group, which suggested that SFPS supplementation induced a more remarkable change in gut microbial structure compared with the HFD group. In the phylum-level analysis, we examined microbial proportions across different groups. SFF and SFPS closely resembled the N group on the clustering tree, but they exhibited high similarity to the HF group, likely due to their shared high-fat diet influence (Fig. 2F). Comparing each group, we noticed distinct changes in microbial composition induced by the high-fat diet. Notably, the high-fat diet decreased the presence of Firmicutes and Cyanobacteria while increasing Actinobacteriota levels.

At the phylum level, the predominant bacterial compositions across all groups included Firmicutes, Verrucomicrobia, Bacteroidetes, Actinobacteria, and Proteobacteria, collectively accounting for over 90% of the relative bacterial abundance. Firmicutes are reduced in both SFF and SFPS groups compared with the HFD group, and the ratio of Firmicutes to Bacteroidetes is reduced in both SFF and SFPS groups.

Obviously, increased abundances of Verrucomicrobia were observed in SFPS-treated mice. The relative abundance of the main microbiota at the genus level in HFD treatment significantly increased the relative abundances of Akkermansia and Faecalibaculum, in contrast to that in the SFPS group. Moreover, the relative abundances of Enterococcus (12.6% vs 41.8%) and Lactobacillus (2.4% vs 14.9%) were decreased by SFPS. (Fig. 2G and H).

As shown in Fig. 2I, we found 43 abundant differential taxa among four groups, including 12 genera. The ND group exhibited a predominance of Lactobacillus, f_Muribaculaceae, s_Candidatus_Arthromitus_sp_SFB_rat_Yit, and s_Lachnospiraceae_bacterium_A2 at the genus level. In contrast, the HFD group featured the genera c_unidentified_Actinobacteria, f_Erysipelotrichaceae, f_Bifidobacteriaceae, and f_Peptostreptococcaceae. Thus, changes in these bacteria might indicate the pathogenesis of HFD-induced disorders. The HFD + SFF group was dominated by o_unidentified_Clostridia, g_Lachnoclostridium, and the HFD + SFPS group was dominated by c_Verrucomicrobiales, p_Cyanobacteria, f_Akkermansiaceae, and p_Proteobacteria, whereas the Akkermansia and Verrucomicrobiales were the most prevalent bacteria at the genus level in the HFD + SFPS group. Thus, these bacteria could serve as biomarkers for SFPS-treated mice and become intestinal indicators to improve HFD-induced hyperlipidemia. Particularly, Akkermansia has been considered a next-generation probiotic (17). Next, the cladogram further demonstrated specific gut microbial taxa related to SFPS treatment.

### SFPS regulates metabolic levels in excreta

**Fig 3:**
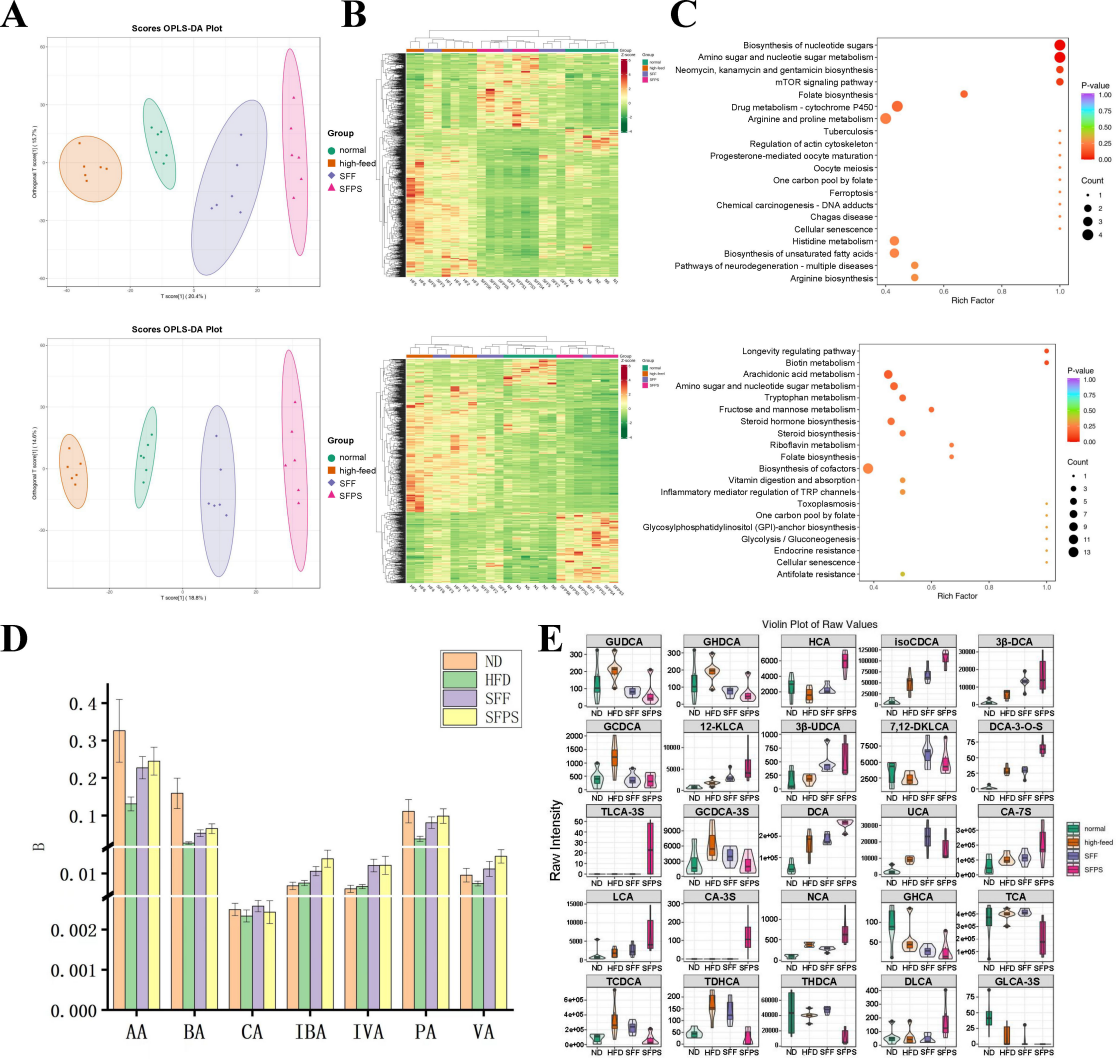
Non-targeted metabolomic profiling of intestinal excreta and differential analysis of short-chain fatty acids and bile acids. (**A**) Orthogonal partial least squares discriminant analysis (OPLS-DA) score chart under positive and negative ion modes. (**B**) Cluster heatmap of differential metabolites screened under positive and negative ion modes. (**C**) Bubble diagram for Kyoto Encyclopedia of Genes and Genomes (KEGG) pathway analysis in positive and negative ion modes. (**D**) Bar chart showing the differential expression of short-chain fatty acids. (**E**) Violin plot of differential expression of bile acids.

Considering the metabolic changes associated with variations in gut microbiota, we initiated LC-MS untargeted metabolic analysis on the four stool sample groups. In our OPLS-DA analysis, distinct groupings of metabolite characteristics were evident in both positive and negative ion modes (Fig. 3A). Heatmap analysis revealed that the differential metabolites identified in the positive ion mode grouped the N, SFF, and SFPS groups together, suggesting that dietary interventions with SFF and SFPS tended to normalize metabolite composition. On the other hand, the differential metabolites identified in the negative ion mode clustered the N and HF groups together, with the primary differences linked to the changes induced by dietary interventions with SFF and SFPS (Fig. 3B).

A further analysis was carried out on the differential metabolites from the N, HF, SFF, and SFPS groups to examine differential metabolic pathways (Fig. 3C). KEGG pathway enrichment analysis in the positive ion mode revealed changes in major metabolic pathways, including folate biosynthesis, nucleotide sugar metabolism, and bile secretion. In the negative ion mode, an intersection analysis of pathways between N vs. HF vs. SFPS and HF vs. SFF showed that the differential metabolites in all groups were primarily associated with fatty acid metabolism, glycerophospholipid metabolism, and amino acid metabolism. This suggests that these metabolic pathways can be altered by SFF and SFPS diets in the context of a high-fat diet, resulting in a regulatory effect on lipid metabolism.

Research has highlighted an interaction between the gut microbiota and the metabolism of short-chain fatty acids (SCFAs) and bile acids. In our non-targeted metabolism studies mentioned earlier, we observed alterations in pathways like bile secretion and fatty acid metabolism. Consequently, we conducted targeted detection of SCFAs and bile acids. Regarding SCFAs, acetic acid (AA), butyric acid (BA), propionic acid (PA), and valeric acid (VA) exhibited a decrease in the HF group, whereas their expression levels were relatively higher in the N, SFF, and SFPS groups. On the other hand, isovaleric acid (IVA) and isobutyric acid (IBA) displayed an increasing trend following SFF and SFPS diets, with no significant differences in the N and HF groups (Fig. 3D). In the context of bile acids, a high-fat diet led to increased levels of GCDCA, GUDCA, GHDCA, and GDCA in the HF group, which were comparatively lower in the N, SFF, and SFPS groups. It is evident that SFF and SFPS can mitigate the elevation of these bile acids. However, the expression levels of 7,12DKLCA, UCA, 12KLCA, 3βDCA, and IsoCDCA were higher in the SFF and SFPS groups. Conversely, GHCA and β-MCA had lower expression levels in the HF, SFF, and SFPS groups due to the influence of the high-fat diet baseline, with SFF and SFPS showing relatively weaker effects (Fig. 3E). Furthermore, a correlation analysis of gut microbiota and fecal BAs was performed. As shown in Fig. 6, Lactobacillus was positively correlated with GHCA, THCA, Tω-MCA, GLCA-3S, GUDCA, GHDCA, GCDCA, THDCA, and TCA, whereas Akkermansia and Verrucomicrobiota were negatively correlated with them. These results further underscore the collaborative regulation of SCFA and bile acid metabolism by both gut microbes and dietary factors.

### SFF and SFPS regulate hepatic lipid metabolism gene expression

**Fig 4:**
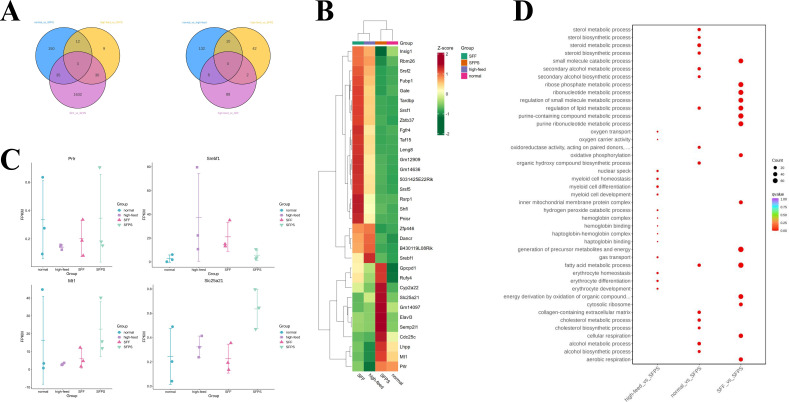
Liver transcriptome analysis. (**A**) Venn diagram analysis of differentially transcribed RNA in the liver. (**B**) Clustering heat map analysis of differentially expressed genes. (**C**) Scatter plot showing the differentially expressed genes in each group. (**D**) Scatter plot of multi-combination Gene Ontology (GO) enrichment.

Having previously explored the link between gut microbes and metabolism, we conducted a liver tissue transcriptome analysis, with a specific focus on genes associated with liver lipid metabolism, to gain a more comprehensive understanding of this mechanism. Three liver samples from each group were subjected to testing and analysis. A differential RNA analysis was conducted, comparing HF vs. SFPS and SFF vs. SFPS, which resulted in the identification of 33 differential RNAs. Further insight was gained by comparing N vs. SFPS, HF vs. SFPS, and SFPS vs. SFF, revealing that 3 RNA substances were unique among the three groups, whereas the remaining 30 showed consistent expression in N vs. SFPS (Fig. 4A). The 33 identified RNAs underwent heatmap analysis to illuminate changes across various expression levels. Genes in Group 1 exhibited higher expression in HF, whereas those in Group 2 displayed lower expression in HF (Fig. 4B). A scatter plot was employed to visualize the specific expression abundance changes of four RNAs (Srebf1, Slc25a21, Prlr, and Mt1), which exhibited statistical differences in each group. Among these, Mt1, Prlr, and Slc25a21 were downregulated in HF and upregulated in N, SFF, and SFPS groups, whereas Srebf1 was upregulated in HF and downregulated in N, SFF, and SFPS groups (Fig. 4C).

Through GO enrichment analysis, the enrichment pathways of differentially expressed substances in N vs. HF, HF vs. SFPS and HF vs. SFF were compared. This analysis revealed that genes differentially expressed in N and HF were primarily enriched in the steroid, sterol, and cholesterol metabolic processes. Conversely, the distinct pathways between HF vs. SFPS and HF vs. SFF was associated with processes such as fatty acid metabolism, carboxylic acid metabolism, and circadian rhythm regulation (Fig. 4D).

### SFPS and SFF regulate plasma lipid metabolism

**Fig 5:**
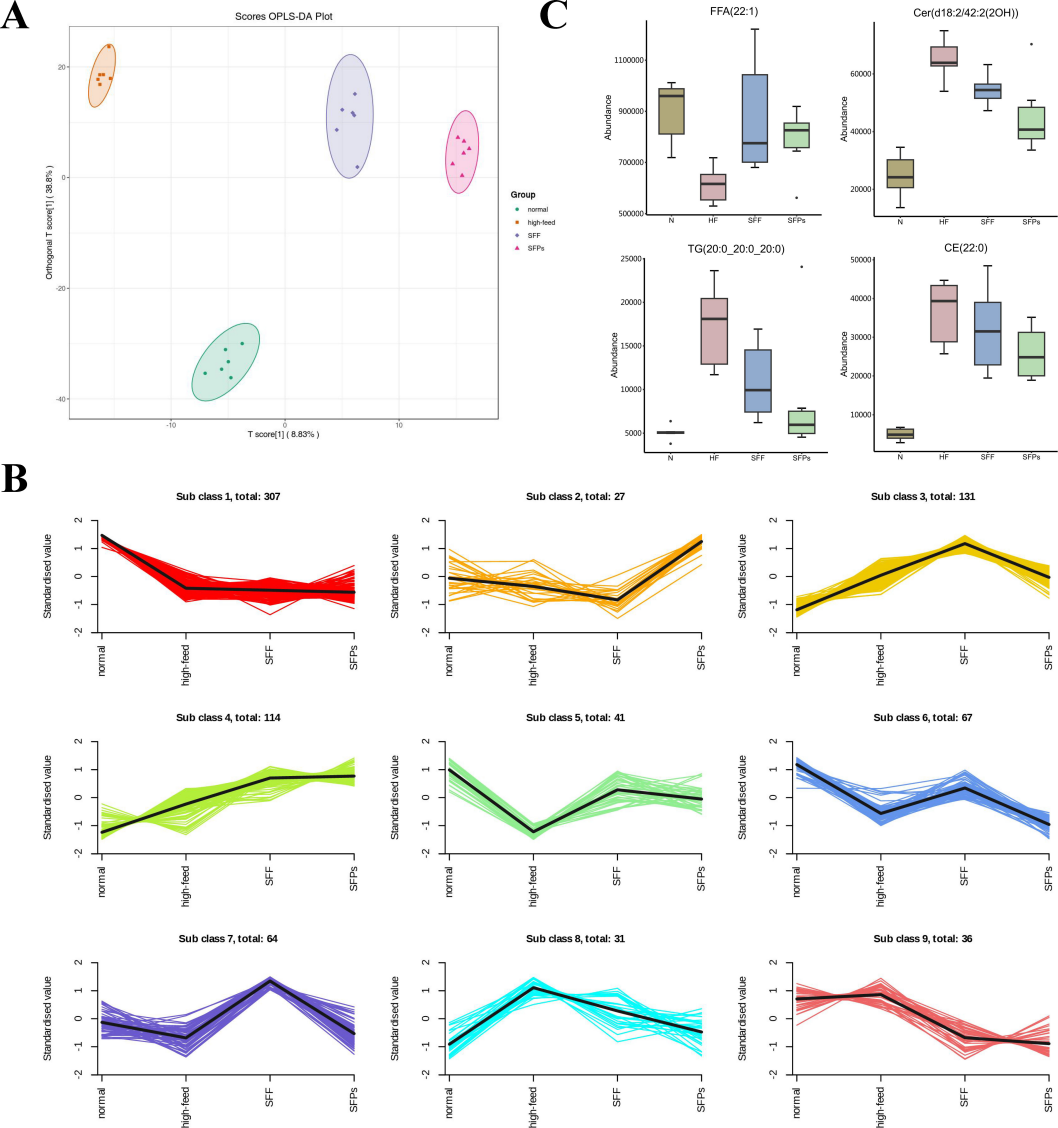
Plasma lipidomics analysis. (**A**) OPLS-DA scores of plasma lipids in N, HF, SFPS, and SFF groups. (**B**) K-means analysis of the lipids in HF, SFPS, and SFF groups. (**C**) Column charts displaying four different lipid species.

Considering our focus on liver gene expression concerning changes in lipid metabolism pathways and to better distinguish specific alterations in plasma lipid metabolism levels, we conducted a comprehensive targeted analysis of plasma lipid metabolism. OPLS-DA analysis demonstrated distinct separation of lipid profiles in each group, highlighting differences in lipid metabolism characteristics (Fig. 5A). K-means analysis revealed that lipids in class 1 displayed a decline in HF, SFPS, and SFF groups, whereas lipids in class 8 were upregulated in HF and remained stable in the N group in SFPS and SFF (Fig. 5B). The lipid composition included cholesterol esters (CE), ceramides (Cer), and TG. Lipids in class 5 were downregulated in HF but remained unchanged in SFPS and SFF compared to the N group. The lipid composition of this class included Cer (t18:0/23:1) and FFA (22:1) (Fig. 5C).

### Multi-omics correlation analysis

**Fig 6:**
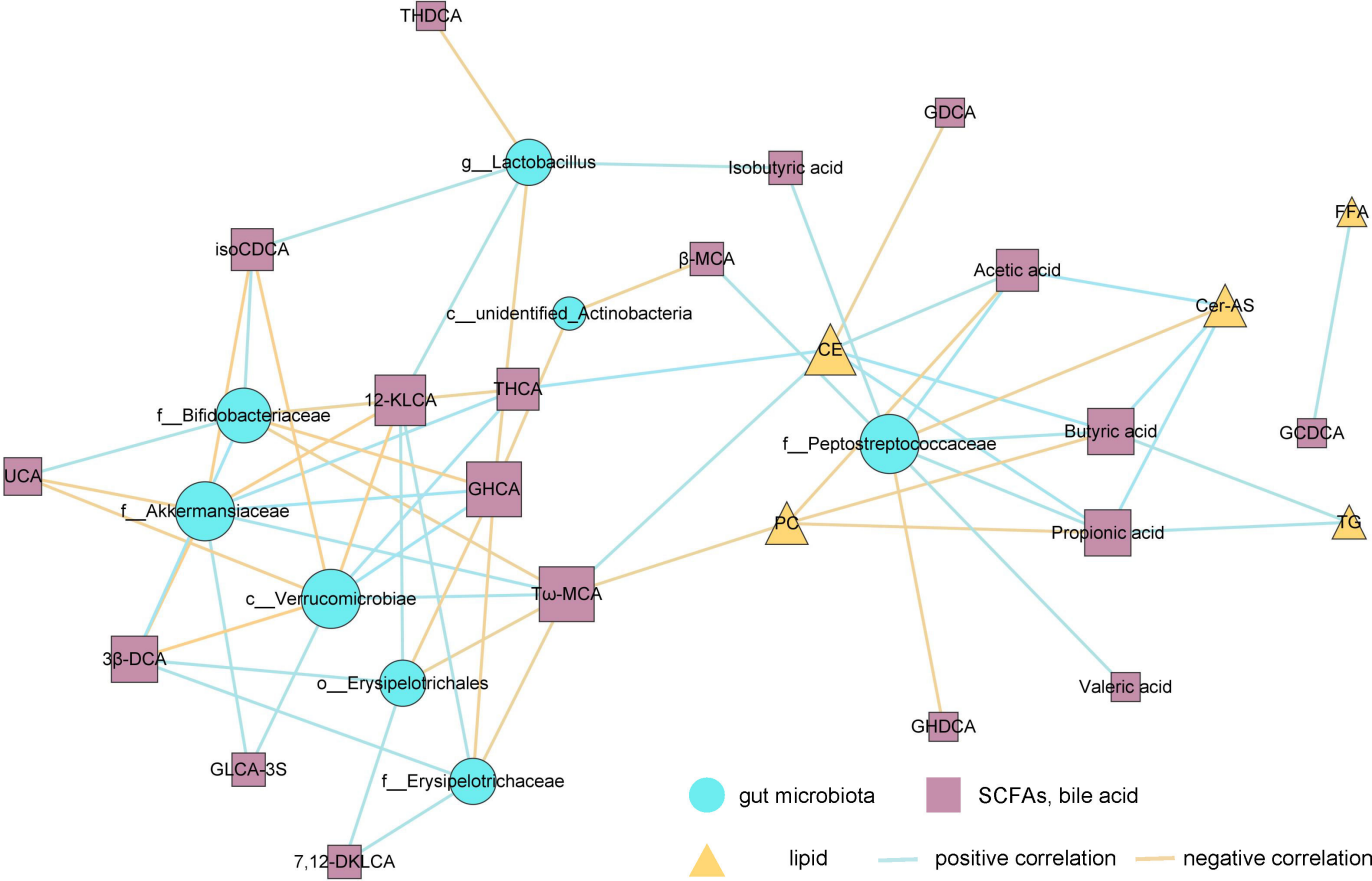
Correlation analysis network diagram depicting relationships between differential gut microbiota, short-chain fatty acids, bile acids, and plasma lipids.

Correlation analyses investigated the relationships between differential microbiota, bile acids, short-chain fatty acid metabolites, and plasma lipid metabolites (Fig. 6). The correlation analysis involved plasma lipids, SCFAs, and bile acids. There is a positive correlation between gut microbiota Akkermansia, Verrucomicrobia, and bile acids UCA, isoCDCA, 3βDCA, and 12KLCA, and a negative correlation with THCA and GHCA. A positive correlation between gut microbiota f_Peptostreptococcaceae and bile acids GHDCA, a negative correlation between gut microbiota f_Peptostreptococcaceae and AA, BA, PA, VA, and IA. Positive correlations were observed between Erysipelotrichaceae and bile acids GHCA and Tω-MCA, and a negative correlation with 7,12-DKLCA,12-KLCA,3β-DCA. Additionally, BA and PA showed correlations with plasma TG, displaying negative correlations. Free fatty acids (FFA) exhibited negative correlations with GCDCA. CE exhibited positive correlations with GDCA and negative correlations with AA, BA, and PA.

**Fig 7:**
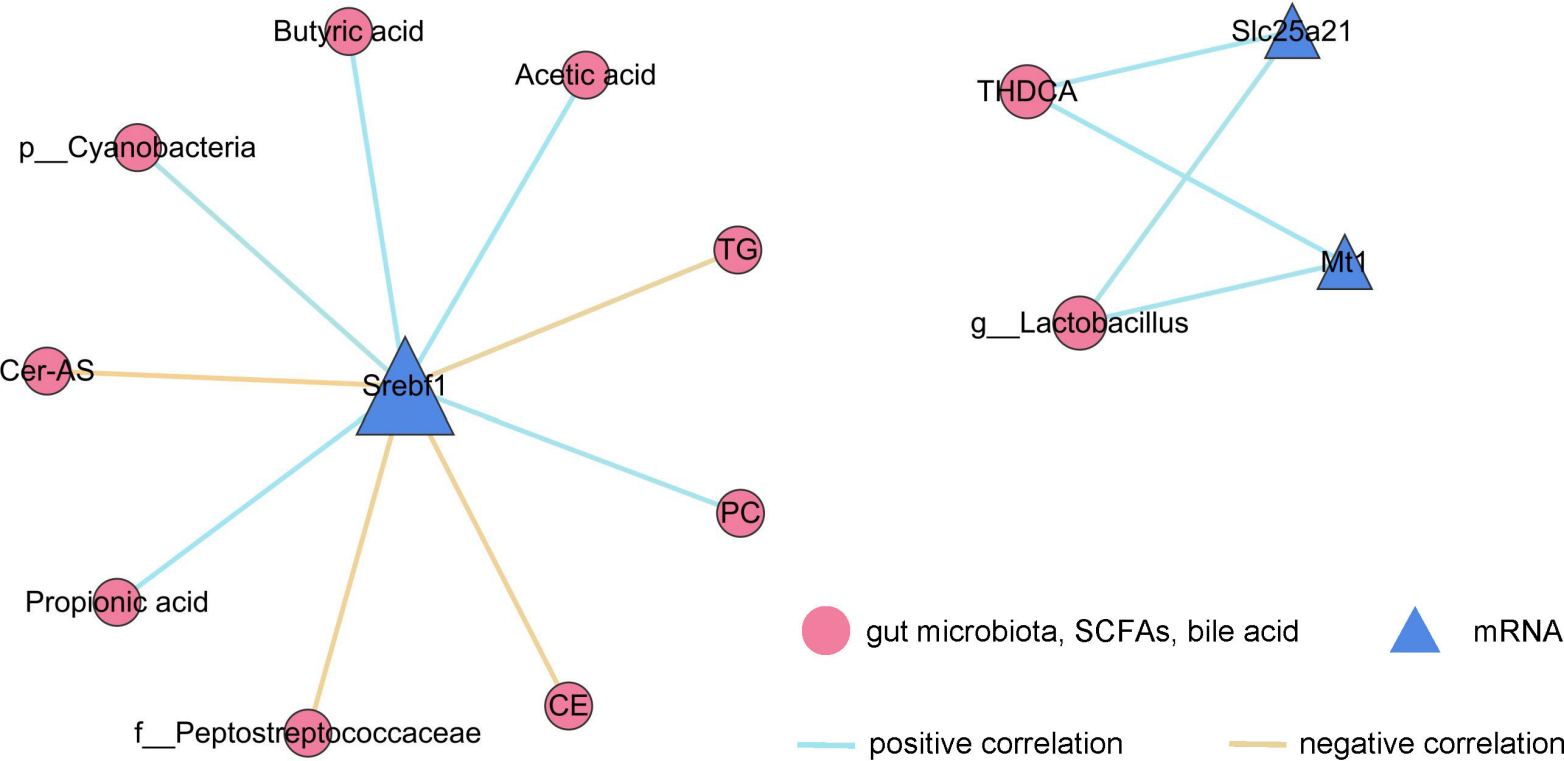
Correlation analysis network diagram illustrating relationships between differentially expressed genes in the liver and gut microbiota, short-chain fatty acids, bile acids, and plasma lipids.

Furthermore, correlation network analysis was performed in conjunction with other omics data for the detected liver transcriptome samples (Fig. 7). Correlations were identified between Srebf1 gene expression and multiple metabolites, including TG, Cer-As, CE, BA, PA, AA, and phosphatidylcholine (PC). Additionally, positive correlations were observed between the Srebf1 gene and the f_Peptostreptococcaceae and a negative correlation between the Srebf1 gene and the p_Cyanobacteria. Slc25a21 and Mt1 demonstrated correlations with bile acids and gut microbiota, displaying a negative correlation with THDCA and g_Lactobacillus.

## DISCUSSION

Long-term intake of a high-fat diet leads to internal metabolic disorders, including disruptions in lipid and bile acid (BA) metabolism, which subsequently induce gut dysbiosis (18). The gut microbiota serves as a crucial mediator between dietary habits and overall health. Given its significant role in lipid metabolism, the potential to alter gut microbial composition through nutritional interventions to mitigate hyperlipidemia has garnered substantial interest in recent years (19). Previous research has demonstrated that fucoidan exhibits antihyperlipidemic effects and modulates gut microbial composition in animals fed an HFD. Consequently, the present study aims to explore whether SFPS can ameliorate HFD-induced hyperlipidemia by regulating the interaction between lipid metabolism and gut microbiota.

This study elucidated the structural characteristics of high-sulfated α-fucoglycans in SFPS through FTIR analysis. This sulfation pattern is closely associated with their potential biological activities. The mode and extent of C4 sulfation influence the negative charge density, thereby modulating the capacity of these polysaccharides to interact with proteins, growth factors, and other biomolecules (20). Furthermore, the configuration of α-glycosidic linkages plays a critical role in determining the three-dimensional conformation, digestibility, and bioactivity of polysaccharides. For example, the α-(1→4) glycosidic bond in amylose—a key component of starch—gives rise to a helical and flexible structure that is well-suited for energy storage. The α-configuration establishes a "*cis*" orientation between adjacent sugar units, favoring chain folding or branching, as observed in highly branched and metabolically accessible polymers such as starch and glycogen (21, 22). Additionally, the presence of trace uronic acid residues may contribute to antioxidant activity via carboxyl group-mediated chelation of metal ions. According to the determination results, it was observed that the polysaccharide content of SFF (92.61% ± 2.41%) was significantly higher than that of SFPS (63.28% ± 3.26%), whereas the protein content of SFF (1.25% ± 0.02%) was considerably lower than that of SFPS (12.41% ± 1.76%). This discrepancy may be attributed to variations in the extraction methods and differences in raw material sources. The protein fraction in SFPS exhibits certain nutritional and biological activities, and its low-molecular-weight component may enhance solubility and bioavailability, potentially explaining the activity differences compared with SFF.

Firmicutes, including Lachnospiraceae, Erysipelotrichaceae, Oscillospiraceae, and Ruminococcaceae, were reported to promote energy resorption and the onset of obesity and diabetes in mice (23, 24). In this study, the HF group exhibited an increase in the F/B ratio, along with a significant reduction in the abundance of Verrucomicrobia and Bacteroidetes. However, SFPS treatment significantly reversed these changes. In the SFF and SFPS groups, g_Faecabiculum, s_FaecabiCulum_rodentium, f_Erysipelotrichaceae, and o_ErsipelotRichales were significantly downregulated. Thus, by targeting these bacteria, SFPS could inhibit the onset of HFD-induced obesity and hyperlipidemia; these microorganisms from different families, genera, and species all belong to the phylum Firmicutes. This study found that a high-fat diet increased the levels of Erysipelotrichaceae and Erysipelotrichale, exacerbating chronic diseases caused by a high-fat diet. The converted sugars in a high-fat diet replace segmented filamentous bacteria by increasing the number of Faecalibaculum rodentium members, thereby increasing the risk of metabolic disorders (4). A previous study reported an elevated Erysipelaceae family in diet-induced obese individuals, which is associated with the regulation of blood lipids (2, 4). Similar to the above studies, the SFF and SFPS diets in this study significantly reduced the proportion of Faecalibacterium in the intestine. Blood lipid levels may be regulated through pathways such as fatty acid degradation, glycerophospholipid metabolism, and primary bile acid metabolism (5).

Akkermansia, recognized as a probiotic, confers positive effects on host health. Recent research has indicated that Akkermansia is instrumental in modulating gut barrier integrity and lipid metabolism *in vivo* (17, 25). It is noteworthy that within the phylum Verrucomicrobia, the LEfSe analysis indicated that Akkermansia emerged as the predominant bacterial genus following the supplementation of SFPS (Fig. 2A and B, Fig. 6). Akkermansia plays a crucial role in safeguarding the host’s intestinal mucosal barrier and maintaining epithelial integrity through the degradation of mucin. Consequently, the inactivation and reduction of Akkermansia may lead to diminished intestinal repair processes and the emergence of hyperlipidemic characteristics (26, 27). Consequently, the elevation of Akkermansia in the group supplemented with SFPS may mitigate the intestinal permeability associated with a high-fat diet by maintaining the integrity of the mucosal layer. Akkermansia, a mucoprotein-degrading bacterium, relies heavily on specific monosaccharides found in mucins, such as fucose, galactose, and N-acetylglucosamine. Studies show that the core monosaccharide composition of fucoidan extracted from Sargassum fusiforme mainly includes fucose (30.6%–55.4%) and galactose (13.1%–24.0%) (27). These components closely match the mucoprotein nutrients needed by Akkermansia, serving as a direct carbon source for its growth. Additionally, *in vitro* fermentation experiments revealed that the levels of acetic acid and propionic acid significantly increased after the gut microbiota degraded polysaccharides from Sargassum fusiforme, consistent with our findings. Akkermansia can use acetic acid—produced during polysaccharide fermentation—as an energy source to support its proliferation (28, 29). We hypothesize that this is one reason why SFPS raises the abundance of Akkermansia.

When exploring the impact of dietary fiber on the gut microbiota, it is not only the composition of fiber that profoundly affects the microbial composition, but also crucial to consider the degradation process of different types of dietary fiber in the intestines (30). Different types of fiber require different microbes to exert their benefits. Through this cooperative enzymatic activity, microbes collectively participate in the breakdown and fermentation of dietary fiber, producing a range of beneficial metabolites such as SCFAs, which positively influence gut and overall health (31, 32). Therefore, understanding the role of SFF and SFPS at the microbial level not only helps unravel the complexity of the gut ecosystem but also provides deeper insights into promoting healthy dietary choices.

During the anaerobic fermentation of dietary fiber by colonic bacteria, acetate, propionate, and butyrate become metabolic byproducts, playing a key role in host metabolic homeostasis (33, 34). These SCFAs, not only being metabolic byproducts, also function as signaling molecules, exerting broad physiological effects by regulating host metabolic processes (35). The studies indicate that SCFAs produced through fiber fermentation in the colon positively regulate glucose and lipid metabolism. Compared with high-fat feeding, an increase in fecal SCFA production may suggest a lower colonic pH, favorable for cholesterol co-precipitation and the removal of unbound bile salts, aiding in cholesterol elimination (36). Additionally, it may regulate blood glucose levels by influencing insulin secretion and insulin resistance, contributing to long-term beneficial effects on overall metabolism. This study observed varying degrees of downregulation of SCFAs in the high-fat diet group, reflecting the adverse impact of a high-fat diet on colonic microbial communities (37), hindering the degradation of dietary fiber and limiting SCFA production. Conversely, in SFF and SFPS rich in dietary fiber, an upregulation trend in SCFAs was observed. This suggests that increasing dietary fiber intake can enhance the ability of colonic microbes to degrade dietary fiber, thereby increasing SCFA production. This upregulation trend may contribute to maintaining or promoting host metabolic health.

Bile acids, pivotal biomolecules synthesized by the liver and excreted into the intestine, serve a crucial role in promoting fat emulsification and absorption (37). Beyond their involvement in lipid metabolism, these compounds play a vital role in regulating metabolic processes by influencing metabolic pathways and reshaping the composition of the gut microbiota. Research underscores that bile acids not only impact lipid absorption but also actively participate in regulating cholesterol homeostasis, exerting a profound influence on host cholesterol metabolism (38). This study delves into the nuanced regulation of bile acid metabolism observed in the SFF and SFPS groups, revealing an intricate interaction with the host gut microbiota. Furthermore, bile acids contribute significantly to maintaining immune balance in the intestine, playing a pivotal role in regulating the intestinal immune system. This regulation occurs through their effect on the integrity of the intestinal mucosal barrier and the activity of immune cells (39).

Sterol regulatory element-binding transcription factors (Srebf), commonly referred to as sterol regulatory element-binding proteins (Srebp), serve as critical transcription factors in lipogenesis. Srebf1 specifically plays a pivotal role in the regulation of lipogenic processes by activating genes associated with the synthesis of fatty acids and TG. Research indicates that dysregulation of lipid metabolism, along with the excessive accumulation of lipids in non-adipose tissues due to the upregulation of srebf1, is implicated in the development of metabolic disorders, including obesity, insulin resistance, and fatty liver disease (40). This study observed that polysaccharides can downregulate the expression of genes such as SREBF1, suggesting that these genes may be involved in the regulation process of their lipid-lowering effect, but their specific regulatory status (such as whether they are key/necessary genes) still needs further verification through subsequent knockout experiments. The expression of the MT1 gene is related to the regulation of lipid metabolism-related genes such as FASN, ACACB, SCD1, etc., which may play a role in reducing blood lipids (41, 42). We observed the down-regulation of Mt1 in the liver of mice in the high-quality diet group, in contrast to the up-regulation of Mt1 in the liver of mice in the SFF and SFPS diet groups. This indicates that SFPS increases the expression of Mt1 and may have potential lipid-lowering functions. In addition, studies have shown that the SLC25A21 gene is associated with high-energy hypolipemia (43); the expression of the Prlr gene is closely related to insulin sensitivity and adipose tissue health (44, 45). Therefore, exploring the relationship between genes and adipose tissue can help us better understand the mechanism of fat metabolism regulation and provide an important theoretical basis for the treatment of obesity and related metabolic diseases in the future.

This study delved into plasma lipidomics, providing a more nuanced exploration of the impact of SFPS and SFF on lipid composition and the molecular signaling of lipid metabolism regulation. Lipidomics is an emerging field that studies lipid metabolism in biological systems. Key lipid metabolic markers in this field include CE, PC, TG, Cer, and fatty acid amides (FAA). Research has found that compared to mice on a normal diet, mice on a high-fat diet exhibit significant differences in the levels of these markers. The significant increase in CE and TG levels indicates a potential link between a high-fat diet and metabolic disorders of cholesterol and triglycerides. The decrease in PC levels suggests that it may be related to impaired phospholipid metabolism in cell membranes. Cer level is increased, suggesting that a high-fat diet may exacerbate inflammatory response and oxidative stress. The imbalance of these metabolic markers ultimately leads to dysregulated lipid metabolism, which in turn can trigger a series of metabolic diseases such as hyperlipidemia. It is suggested that supplementation with SFPS and SFF will help alleviate the levels of these indicators. Therefore, by modulating these key lipid metabolic markers, there is hope to provide new targets and strategies for the prevention and treatment of related diseases.

Through correlation analysis, our study reveals that SFPS and SFF may decrease the abundance of f_Peptostreptococcaceae, f__Erysipelotirichaceae, and o__Erysipelotrichales and increase Akkermansia and c_Verrucomicrobia, impacting the breakdown of fiber in *Sargassum fusiforme* polysaccharides. This process leads to the production of SCFAs like AA, BA, PA, and VA, potentially elevating their levels. Consequently, this may enhance the cyclic reabsorption of bile acids, including GDCA, GUDCA, GHDCA, and GHCA, while also influencing the excretion of 7,12DKLCA, UCA, 12KLCA, 3βDCA, and IsoCDCA.

The integration of liver transcriptome data with multi-omics approaches has unveiled a complex regulatory network. The expression of Srebf1 (sterol regulatory element-binding transcription factor 1) is closely associated with various lipid-related metabolites, including triglycerides (TG), ceramides (Cer-As), cholesterol esters (CE), bile acids (BA), phosphatidic acids (PA), arachidonic acids (AA), and phosphatidylcholine (PC). The strong correlation between PC and Srebf1 suggests that SREBF1 plays a central role in hepatic lipid metabolism and the maintenance of systemic metabolic homeostasis (46). As a key regulator of adipogenesis, Srebf1’s involvement aligns with its observed association with lipid metabolites within this network (47). Moreover, the correlation between Slc25a21 and Mt1 highlights the interplay among mitochondrial function, oxidative stress, and bile acid homeostasis. The negative correlation between Slc25a21 (a mitochondrial transport protein involved in fatty acid oxidation) and THDCA (taurine-conjugated deoxycholic acid) indicates the presence of a potential feedback regulatory mechanism in bile acid metabolism (48, 49).

In summary, our results showed that SFPS and SFF exhibited promising antihyperlipidemic effects in HFD-fed mice by regulating the BA metabolism and gut microbiota. Eight weeks of SFPS supplementation significantly attenuated the hyperlipidemic syndromes in HFD-fed mice. Besides, SFPS and SFF significantly upregulated the expression of Mt1, Prlr, and slc25a21 and downregulated the expression of SREBP-1; therefore, the lipogenesis was restrained; meanwhile, lipolysis, BA synthesis, and efflux to feces via the Cholesterol metabolism pathway were improved. Also, SFPS and SFF markedly increased the abundance of probiotics, such as Akkermansia and Verrucomicrobia, while decreasing the abundance of hyperlipidemia-related bacteria, such as c_unidentified_Actinobacteria, f_Erysipelotrichaceae, f_Bifidobacteriaceae, and f_Peptostreptococcaceae; thus, SFPS improved gut dysbiosis caused by HFD via the activation of the cholesterol metabolism pathway. SFPS could serve as a promising functional ingredient for hyperlipidemic people to improve public health.

This study also has some limitations, such as a small sample size and being mainly based on mouse models. Further in-depth research is needed to extend these research results to the human body. Current research has revealed the potential mechanism by which SFPS improves hyperlipidemia through multi-omics (microbiota, metabolites, genes), but due to limitations such as one-way intervention design, small sample size, and complex polysaccharide components, the evidence chain of the causal relationship of the mechanism is still incomplete. In the future, it is necessary to fully develop the potential of SFPS as a functional food ingredient for anti-obesity through reverse validation experiments, active ingredient analysis, in-depth cross-omics integration, and the promotion of clinical translational research.

## Data Availability

Supplementary materials, including diversity analysis results, NMDS raw data, and liver transcriptome data, are archived in ScienceDB (https://doi.org/10.57760/sciencedb.28108). Additionally, the original data sets (comprising a subset of 16S rRNA gene sequencing data, liver transcriptome data, and targeted metabolomics data) are available at https://doi.org/10.57760/sciencedb.29696.

## SUPPLEMENTAL MATERIAL

Figure S1 aem.01445-25-s0001.docx Analysis of SFPS using FT-IR spectra.

ASM does not own the copyrights to Supplemental Material that may be linked to, or accessed through, an article. The authors have granted ASM a non-exclusive, world-wide license to publish the Supplemental Material files. Please contact the corresponding author directly for reuse.
