# Supplementary material for: Sargassum fusiforme polysaccharides modulate gut microbiota and metabolites to regulate hyperlipidemia in mice fed a high-fat diet
Source: Appl Environ Microbiol. 2025 Nov 11;91(12):e01445-25. doi: 10.1128/aem.01445-25 (PMC12724339; doi:10.1128/aem.01445-25)
Supplement: Figure S1 — Analysis of SFPS using FT-IR spectra. [file aem.01445-25-s0001.docx]

*Sargassum fusiforme* polysaccharides modulate gut microbiota and metabolites to regulate hyperlipidemia in mice fed a high-fat diet

Ning Su^1^,Shiwei Han^1^,Zhengyang Li^1^,Xiaoyu Ling^1^,Lingqing Kong^1^,Dafeng Song^1*^


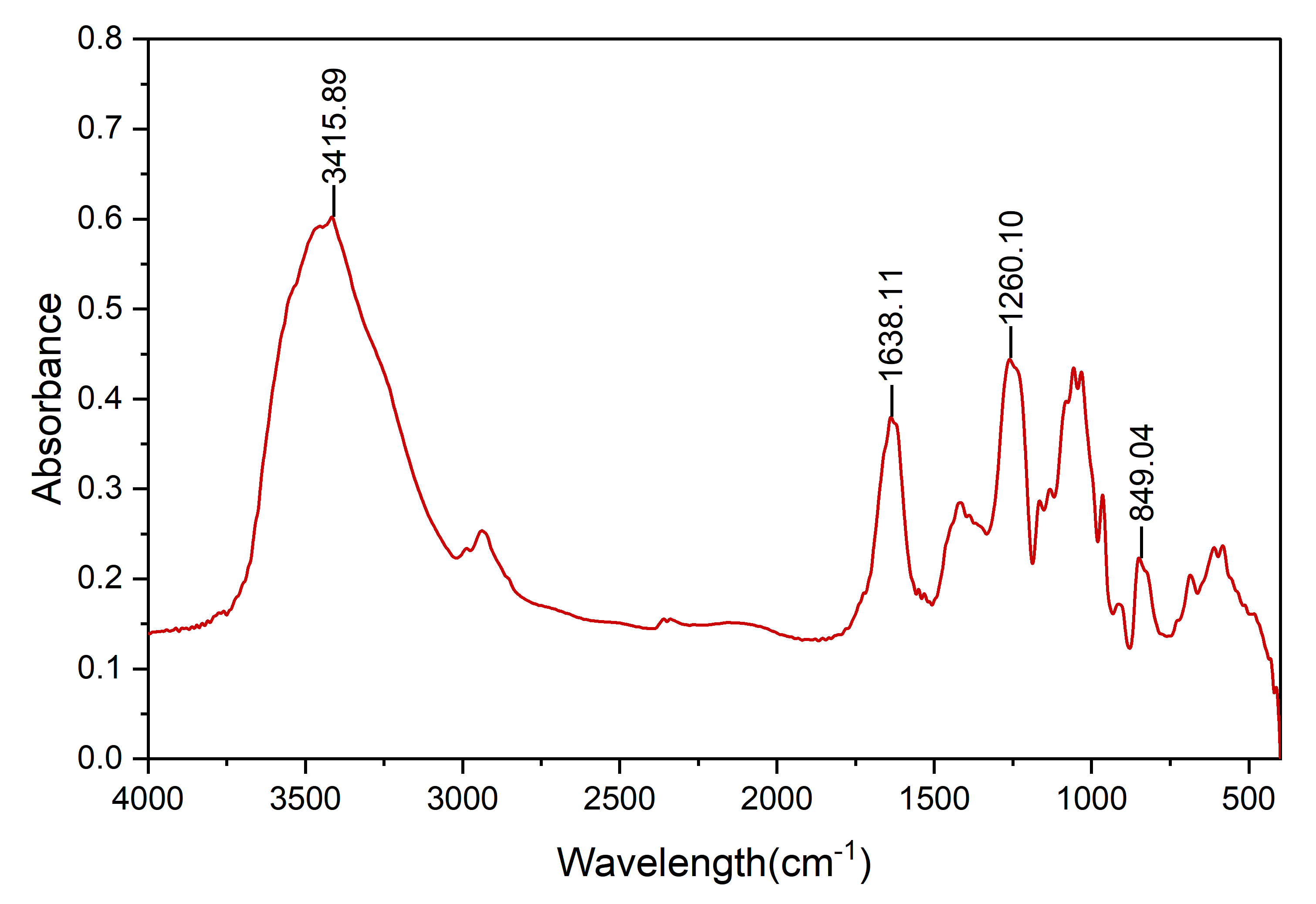


**Supplementary Figure1.** Analysis of SFPS using FT-IR spectra.
